# Membrane–Peptide Interactions: From Basics to Current Applications 2.0

**DOI:** 10.3390/ijms24087202

**Published:** 2023-04-13

**Authors:** Sónia Gonçalves, Nuno C. Santos

**Affiliations:** Instituto de Medicina Molecular, Faculdade de Medicina, Universidade de Lisboa, 1649-028 Lisbon, Portugal

The interaction between peptides and biological membranes is of fundamental importance in the mechanism of numerous membrane-mediated cellular processes, including antimicrobial peptide action, hormone–receptor interactions, drug bioavailability across the blood–brain barrier, and viral fusion processes. In this second Special Issue, peptides obtained from different sources (e.g., animals [1], humans [2], and in silico designed [3,4]) were used as potential therapeutic molecules, mostly for human health applications. This Special Issue, Membrane–Peptide Interactions: From Basics to Current Applications 2.0, includes a selection of five original research articles exploring the determinants of peptide–membrane interactions. As in the first edition, authors brought into play biological approaches, together with biophysical methodologies, to understand peptide–membrane interactions.

Studies with lipid systems mimicking microbial membranes, together with in vitro assays with cells, have been essential for studying the mode of action of different molecules at the membrane level. This way, three works extensively explored peptide–membrane interactions [1,2,3]. In the first article, de Aguiar et al. [1] presented a detailed study of the antimicrobial activity of Ctn[15–34]. Combining experimental techniques such as confocal laser scanning microscopy and atomic force microscopy (AFM), the authors assessed the antibiofilm effect of Ctn[15–34] against *Candida albicans*. Membrane assays through the use of the fluorescent probe di-8-ANEPPS, dynamic light scattering, and zeta potential measurements using liposomes, protoplasts, and *C. albicans* cells indicated a direct mechanism of action dependent on membrane interaction and its disruption. Ctn[15–34] was shown to be an effective antifungal peptide, displaying antibiofilm activity by disrupting fungal plasma membranes.

In the work developed by Ambrosio et al. [3], the authors showed the antimicrobial activity of the peptide 1018-K6 (VRLIVKVRIWRR-NH_2_) against *Listeria monocytogenes* and *Salmonella* spp. biofilms. Moreover, to understand the relationship between the peptide–membrane interactions and their activity, this study combined model membranes, either purely synthetic or prepared with lipids extracted from bacteria. Using conventional biophysical methodologies such as fluorescence quenching, membrane leakage, and lipid sedimentation assays, the authors determined that the peptide 1018-K6 specifically binds to bacterial but not to eukaryotic cell membranes. Its antibacterial mechanism of action seems to be associated with its binding and mode of interaction with different lipid bilayers.

A different experimental work with lipid vesicles was developed by Farilé-Águeda et al. [2]. They studied the synergism between two lung proteins with antimicrobial activity against *Klebsiella pneumoniae*. Surfactant protein A (SP-A) and the N-terminal segment of the SP-B pro-protein (SP-B^N^) are important human lung defense antimicrobial molecules. In Farilé-Águeda et al.’s work, membrane permeabilization and depolarization assays, as well as transmission electron microscopy, were used to study the effect of these proteins and peptides on *K. pneumoniae*. Their effect on model membranes of outer and inner bacterial membranes was analyzed by differential scanning calorimetry and membrane leakage assays. Their results indicate that the SP-A/SP-B^N^ complex alters the ultrastructure of *K. pneumoniae* by binding to the lipopolysaccharide molecules present in the outer leaflet of the outer bacterial membrane, inducing membrane packing defects that could favor the translocation of both molecules to the periplasmic space. The SP-A/SP-B^N^ complex also depolarized and permeabilized the inner bacterial membrane, probably through the formation of toroidal pores. Overall, the synergistic antimicrobial activity of SP-A and SP-B^N^ is based on the ability of this complex, but not on each one of its components alone, to impair the integrity of bacterial membranes.

Contrary to conventional antibiotics, antimicrobial peptides (AMPs) are able not only to show a broad-spectrum activity against microorganisms, but often, at the same time, display the ability to neutralize toxins, modulate the inflammatory response, eradicate bacterial and fungal biofilms, or prevent their development. Some antimicrobials have a limited ability to diffuse through the rigid structures of biofilms. AMP lipidation can be considered an effective approach for the enhancement of their antimicrobial potential and in vivo stability; however, it may also have an undesired impact on selectivity, solubility, or the aggregation of the modified peptides. Kosikowska-Adamus et al. [4] describe the results of the structural modifications of compounds that are designed based on the cationic antimicrobial peptides DK5 and CAR-PEG-DK5, derivatized at their N-terminal with fatty acyls of different lengths. These modifications improved the antimicrobial properties of the final compounds and their effectiveness in inhibiting biofilm development, as well as in the eradication of pre-formed biofilms of *C. albicans* and *Staphylococcus aureus*. Molecular dynamics simulations revealed a strong correlation between the length of fatty acid conjugated with the peptide and the conjugate’s membranolytic properties, tendency to self-assemble and cytotoxicity. In general, after insertion, the peptides were dispersed throughout the membrane, arranged with their α-helical axis parallel to the membrane surface. Peptide binding resulted in a local increase in the apparent area per lipid in the outer leaflet of the membrane, which suggests the disintegration of the membrane and can be related to the antimicrobial efficacy of the lipopeptides. This study is a great example of how experimental and in silico methods can complement each other.

Continuous improvements in quantum mechanical calculations (QMC) have led to increased applications in biological problems. Papp et al. [5] developed an elegant work based on QMC to study the effect of bulk protein environments. The authors’ research provides insight at the atomic level into structural and energetic properties of the mechanical unfolding of small peptide models under the influence of two bulk environments: the aqueous (W) and a membrane-like liquid protein phase (LP). Two models employed lateral unfolding and sheared unfolding to investigate the mechanisms of an extended β-sheet model of 22 alanine residues. Based on the QMC presented, W and LP environments were shown to have a strong effect on the folding properties and affinity of protein and peptide segments. The authors’ intention was to help to understand the LP phase of membraneless organelles and the lipophilic interior of biomembranes, providing quantitative information on why an inner separated hydrophobic region is required for chaperones to drive protein folding more effectively.

In the first edition of *Membrane–Peptide Interactions: From Basics to Current Applications*, we attempted to provide a collection of original research where the main focus was the characterization of peptide-membrane systems through the use of practical approaches. Following the same line of thought, we have moved on to this second edition. We hope this expansion of the previously focused topics to be helpful for many readers.
